# Predictors of dopamine agonist resistance in prolactinoma patients

**DOI:** 10.1186/s12902-020-0543-4

**Published:** 2020-05-19

**Authors:** Elle Vermeulen, Jean D’Haens, Tadeusz Stadnik, David Unuane, Kurt Barbe, Vera Van Velthoven, Sven Gläsker

**Affiliations:** 1 Department of Neurosurgery, VUB University Hospital of Brussels, Laarbeeklaan 101, 1090 Brussels, Belgium; 2 Department of Radiology, VUB University Hospital of Brussels, Laarbeeklaan 101, 1090, Brussels, Belgium; 3 Department of Endocrinology, VUB University Hospital of Brussels, Laarbeeklaan 101, 1090, Brussels, Belgium; 4 Department of Statistics, VUB University Hospital of Brussels, Laarbeeklaan 101, 1090, Brussels, Belgium

**Keywords:** Pituitary adenoma, Prolactinoma, Resistance, Dopamine agonist

## Abstract

**Background:**

Surgical resection of prolactinomas resistant to dopamine agonists is frequently incomplete due to fibrotic changes of the tumour under pharmacological therapy. In order to identify a subgroup of patients who may benefit from early surgery, we thought to investigate possible predictive factors of pharmacological resistance of prolactinomas to dopamine agonists.

**Methods:**

We retrospectively analyzed a database of a Belgian tertiary reference center for patients with pituitary tumours from 2014 to 2016. The groups of interest were patients with dopamine agonist responsive and resistant prolactinomas. The possible predictive factors, including MRI findings, endocrinological parameters, response of tumour and patient factors for dopamine agonist resistance were investigated.

**Results:**

We included 69 patients of whom 52 were women (75,4%) and 17 were men (24,6%). Rate of dopamine agonist resistance was 15.9%. We identified four significant predictors of dopamine agonist resistance: male gender, a large tumour volume, prolonged time to prolactin normalization and presence of a cystic, hemorrhagic and/or necrotic component. In addition, symptoms due to mass effect, high baseline prolactin level and a high contrast capture on MRI are factors that can be taken into consideration.

**Conclusion:**

We identified predictive factors for pharmacological resistance and developed a scoring system for patient specific prediction of resistance to dopamine agonists. This scoring system may have impact on the timing and decision of surgery in prolactinoma patients after further prospective evaluation.

## Background

Pituitary adenomas are benign neuro-endocrine tumours and represent 10% of all intracranial tumours. The most common hormone-secreting pituitary tumours are prolactinomas, accounting for approximately 40% [1–3]. Prolactin (PRL) secreting adenomas are particular in the responsiveness to a pharmacological therapy in contrast to other pituitary tumours [1].

Dopamine reduces the secretion of prolactin and tumour volume by its suppressive effect on lactotrophic cells in the pituitary and by lowering the angiogenesesis in the surrounding tissue [1, 4]. The first-line treatment of prolactinomas with dopamine agonists (DA) is based on this mechanism [5]. A minority (5–18%) of patients treated with dopamine agonists, nowadays mainly using cabergoline (CAB) instead of the older variant bromocriptine (BRC), do not achieve sufficient response. This is most commonly owed to resistance or intolerance [2].

At present, there is no universal definition of dopamine agonist resistance. However, considering the possible detrimental effect of hyperprolactinemia as well as tumour volume, a reasonable definition is regarded as the failure to achieve prolactin normalization and/or a tumour size reduction in coronal surface of ≥50%. The definition only applies after a minimum period of 3 months of receiving a daily dose of 15 mg bromocriptine or a weekly dose of 3.0 mg of cabergoline if tolerated [2]. Dopamine agonist resistance occurs in patients with micro- and macroprolactinoma, in 5 and 20% respectively [2, 3, 6, 7]. Despite the fact that the side effects of DA are limited, the intolerance rate is estimated to be approximately 3–12% [2, 8]. Drug resistance and intolerance, together with patient’s preference, diagnostic uncertainty and complications such as tumour apoplexy, visual impairment and cerebrospinal fluid (CSF) leakage due to shrinkage of the tumour are indications for trans-sphenoidal surgery [8]. The remission rate of surgery is approximately 73–90% in microprolactinoma cases and 33–56% in macroprolactinoma cases [3, 8]. The side effects of surgery are rare and can be divided in two groups: the minor (3.5–6.5%, e.g. septal perforation, epistaxis, wound infection, hematoma, CSF leak and diabetes insipidus) and major (1.5%, e.g. vascular injury/stroke, meningitis/abscess, visual loss and oculomotor injury) complications [2, 8].

When resistance (failure to achieve PRL normalization and/or a tumour size reduction of ≥50%) is established to a particular patient, there are different therapeutic options [9]. One of the options is to switch to another DA since there is clear evidence that the switch to cabergoline can overcome resistance to bromocriptine, with a normalization of PRL and tumour mass reduction in 80 and 70% of the cases respectively [3, 6, 8]. Another approach is a step-up dose augmentation if tolerated (side-effects are too prominent) and given a continued response. Although partial resistance may be suppressed by a gradual increase of the dosage of dopamine agonists, it seems that for cabergoline (regarded as the most efficient dopamine agonist) there is little benefit when increasing the dose above 3.0 mg per week if continued for at least 3–6 months [6, 10]. Another option is transsphenoidal surgery, which is widely considered as the next gesture in cases of DA resistance.

However, second line surgical resection of dopamine agonist resistant prolactinomas is frequently incomplete because of fibrotic changes of the tumour due to dopamine agonists [11, 12]. Fibrosis and uneven shrinkage of the tumour can establish after 6 weeks of bromocriptine treatment [11, 12]. A recent study showed that 77% of the prolactinomas with bromocriptine pretreatment were fibrotic. The probability of fibrosis (22%) after 1 month cabergoline treatment was lower but still present [13]. Finding fibrosis at the time of surgery is considered as a negative predictive factor for complete biochemical remission after surgery (0% versus 37%) [1, 8, 13, 14]. As a consequence, the overall prolactin remission rate after second line surgery in patients with prolactinoma not-responding to dopamine agonists, is approximately 36% [4, 8]. This is a considerably lower remission rate compared to about 87% in microprolactinoma and 56% in macroprolactinoma after first-line surgery, before the fibrotic changes can occur. In addition, complications such as diabetes insipidus are significantly more frequent as a post-operative complication in patients with bromocriptine pretreatment compared to non-pretreated prolactinomas [1]. Therefore, identifying the subgroup of patients with high risk of dopamine agonist resistance may be important since they could benefit from surgery early on in the course of the treatment given the better remission and complete resection outcome [7, 12]. In order to identify such subgroups of patients, we thought to investigate predictive factors of resistance to dopamine agonists in a consecutive series of prolactinoma patients treated at our hospital.

## Methods

### Clinical data

To identify predictive factors of dopamine agonist resistance, we conducted a retrospective study based on a database of patients treated in a Belgian tertiary reference center from 2014 to 2016 after approval of the ethical commission of our hospital. From the moment of the start of the study, we went back in time to when a sufficient number of patients, determined in a power analysis, could be included.

Consent has been obtained from each patient after full explanation of the purpose and nature of all procedures. The inclusion criteria of the patients for this study were: age of 18 years or older, a confirmed prolactinoma and an available MRI before the start of therapy. The diagnosis of prolactinoma was based on the assessment of our endocrinologists. Here, clinical presentation, exclusion of other causes of hyperprolactinemia, MRI imaging and laboratory findings were taken into account. We considered the diagnosis of prolactinoma only in the presence of a corresponding MRI image and prolactin levels that were clearly elevated corresponding with levels at least 2 times higher than the upper limit of normal for microadenomas (except in one case) and at least 5 times higher than the upper limit of normal for macroadenomas and established on two separate occasions.

Patients with familial tumour syndromes such as multiple endocrine neoplasia type 1 (MEN1) were excluded.

Clinical data with possible predictive relevance were extracted from the patient’s records: sex, age, age at diagnosis, presence of sexual dysfunction (amenorrhea or oligomenorrhea, infertility, decreased libido, erectile disorder) or galactorrhea, presence of mass effects and the duration of the symptoms before diagnosis. Mass effects disembosom: headache, dizziness, visual defects (abnormal visual field or eye movement disorder) and cranial neuropathy.

The biochemical data we determined, were the levels of PRL, insulin like growth factor -I (IGF-I), thyroid stimulating hormone (TSH) and adrenocorticotropic hormone (ACTH) and we quoted the presence of sex hormone deficiency defined as decreased gonadotropins or testosterone.

Furthermore we examined biochemical data as well as different MRI variables such as the volume of the tumour (h x l x w x (π/6)); the shape of the tumour (being a sphere or bifocal); the intensity factor which is calculated as the ratio between the intensity of the tumour and the intensity of the grey matter (displayed in pixel value, PV), always consequently at the level of the superior temporal gyrus. Contrast enhancement, which is the ratio of the density factor on T1 after contrast to the value on T1 without contrast, was likewise computed. We also investigated parameters for follow up regarding the evolution of the tumour volume and the prolactin level.

The dosage of cabergoline was monitored during treatment in order to evaluate the response.

The dopamine agonist dosing regimen was clinically adjusted and examined as follows. Effect of DA treatment was re-evaluated at least every 3 months. If the treatment goal was achieved in terms of prolactin level and tumour size, that same dose would have been continued. If treatment goal was not achieved, the dose would be augmented after evaluation by the endocrinologists at the consultation.

When surgery was involved, a histological examination of the surgical specimen was always performed. Information about the Ki 67 (proliferation marker) level and the presence of sclerosis was hereby obtained. Sclerosis (augmentation of fibrous tissue) is considered negative when there was no mention of fibrosis, connective tissue or sclerosis in the operation report; and when it is not defined by the anatomy pathologist in the histological examination report of the surgical specimen.

Patients assigned to the responsive group had both prolactin normalization and a tumour volume shrinkage of ≥50% in coronal surface under dopamine agonist treatment. Resistance was concluded if no hormonal or tumoural response could be achieved after the next 1–2 consultations with a weekly dose up to 3.0 mg cabergoline (see definition).

However, in that case, if tolerated and with the patient consent, the dosage would further be increased. The response was monitored throughout the follow-up (at least 12 months) and changed if there was a response after that further dose escalation.

Thereafter; a comparative study between the 2 groups, responsive versus resistant prolactinoma patients was performed.

### Statistical analysis

The statistical analysis was performed using IBM SPSS statistics subscription software (International Business Machines Corp., Armonk, New York, USA). The level of significance was set at *P* < 0.05. First, an exploratory analysis was performed wherein we examined for all factors whether there was a significant difference between the group of patients resistant to the dopamine agonists and the group that responds well to this first-line treatment. Chi-squared test (χ^2^ test) was performed to compare count data. Wilcoxon-Mann-Whitney test and t-test were performed to compare continuous data. The aim of this explorative statistical phase was to select the most promising predictive factors to include in the second statistical analysis in order not to overfit the proceeding analysis which would lower the predictive power. In the second confirming statistical phase, a Fisher’s linear discriminant analysis was used to quantify the contribution of all studied parameters in the prediction of possible resistance to the dopamine agonist cabergoline. The factors which are included in this analysis were selected on the basis of a (borderline) significant difference between the 2 groups (in the first statistical phase) and/or a correlation with resistance detected in previous literature. The ultimate goal was to develop a scoring system for practical use in a clinical setting to assess resistance.

## Results

### Patient population

A total of 69 patients of whom 52 women (75.4%) and 17 men (24.6%), were included in the study. There was a fairly balanced ratio between the overall prevalence of micro- and macroprolactinoma in our study population, 54.4 and 45.6% respectively (Table 1). However, there was a higher occurrence of macroprolactinomas in men compared to women (88.2% in men versus 30.8% in women). The median baseline prolactin level, before start of the therapy, was 116.98 μg/l (interquartile range, IQR = 294.46 μg/l), with a lowest value of 26.03 μg/l and a highest of 4488.73 μg/l.

**Table 1:** Descriptive parameters of the total study population

		**Study population**	**Responsive group**	**Resistant group**
Demographic factors	**Patients**	69	58	11
	**Women**	52	46	6
	**Men**	17	12	5
Endocrinological factors	**Baseline PRL** **[median (interquartile range)]**	116.98 μg/l (294,46)	105.66 μg/l (284,17)	259.77 μg/l (842,24)
	**PRL after 3 months: Reduction** **[mean (standard deviation)]**	83.3% (20.56)	85.9% [11, 15]	67.5% (31,75)
	**PRL after 3 months: Normalization**	62.0%	70.5%	0.0%
	**PRL after 4 months: Reduction** **[mean (standard deviation)]**	82.7% (22,85)	87.4% (18,95)	63.8% (30,32)
	**PRL after 4 months: Normalization**	79.0%	87.5%	0.0%
MRI factors	**Tumour volume** **[median (interquartile range)]**	0.18 cm^3^ (1,32)	0.13 cm^3^ (0,88)	3.34 cm^3^ [5, 7]
	**Microadenoma**	54.4%	59.6%	27.3%
	**Macroadenoma**	45.6%	40.4%	72.7%
	**Contrast capitation** **[mean (standard deviation)]**	1.93 (0,81)	1.88 (0,70)	2.40 (1,43)
	**Presence of cystic/hemorraghic/necrotic component**	26.7%	20.7%	71.4%

PRL: Prolactin; Contrast capitation: the ratio of the density factor on T1 after contrast to the value on T1 without contrast

Of the 67 included patients, 61 patients (91%) were treated with cabergoline and 4 patients (6%) with bromocriptine. In 2 patients (3%) there was a switch from one dopamine agonist to another during the course of our study. The median dose of cabergoline in the total study group was 0.5 mg/week with an interquartile range of 0.75 mg/week. Transsphenoidal resection of the adenoma was performed in 9 patients, representing 13% of the study population. In two patients, surgery was performed as first-line treatment due to patient preference or compression of the optic chiasm. Resistance to dopamine agonist was the underlying cause for second line surgery in 6 out of 7 patients. One patient was intolerant to the dopamine agonists. Histological analysis of the surgically resected tissue confirmed the diagnosis in all cases. In the 2 patients who underwent early surgery, there was neither sclerosis nor an elevated Ki67 level. The Ki 67 level of patients who underwent surgery after DA pretreatment was increased in 3 out of 4.

Resistance to dopamine agonists is seen in 11 of the 69 patients, representing 15,9% of our total study population.

### Dopamine agonist responsive versus resistant patients

The total study population was divided into 2 groups. The first group consists of 58 patients that had sufficient response to dopamine agonist (= responsive group). The remaining 11 patients pertain the resistant group (Table 2). Causes of resistance in this second group were: absence of prolactin normalization (5/11), < 50% tumour volume shrinkage (1/11) or the failure of both hormonal and tumour response (5/11). The demography of the 2 groups was mainly different in terms of gender with more men found in the resistant group compared to the responsive group (45.5% vs. 20.7% respectively; *p* = 0.08).

**Table 2:** Descriptive parameters of the resistant patients

Sex	Baseline PRL (μg/l)	Age at diagnose (range in years)	Symptoms (*)	Mass effects	Tumour classification	Tumour volume (cm^3^)	Cystic/ Necrotic/ hemorragic component	Surgery	Sclerosis
Man	668.67	20–30	Sexual dysfunction	Headache/ Dizziness	Macroadenoma	3.34	Yes	Yes	Yes
Woman	126.9	20–30	Menstrual disturbances	None	Macroadenoma	3.33	Yes	No	/
Woman	77,2	20–30	Menstrual dysfunction + galactorrhea	Visual defects	Microadenoma	Not known	Not known	Yes	No
Man	1058.3	50–60	None	Headache/ Dizziness	Macroadenoma	6.93	Yes	Yes	Yes
Man	230.65	60–70	Sexual dysfunction	Headache/ Dizziness	Macroadenoma	2.041	No	No	/
Man	253.77	30–40	Sexual dysfunction	None	Macroadenoma	1.23	Yes	Yes	Yes
Man	332.43	20–30	Menstrual dysfunction	None	Macroadenoma	41.36	No	Yes	Yes
Woman	2582.3	60–70	None	Visual defects	Macroadenoma	2.65	No	No	/
Woman	131.0	50–60	Menstrual dysfunction	None	Microadenoma	Not known	Not kown	Yes	Yes
Woman	90.19	40–50	Menstrual dysfunction	None	Macroadenoma	Not known	Yes	No	/
Woman	162.0	20–30	Menstrual dysfunction + galactorrhea	None	Macroadenoma	Not known	Yes	No	/

(*) Sexual dysfunction: Decreased libido + Erectile disorder; Menstrual disturbance: Amenorrhea + Infertility

Although, the presence of symptoms was evenly distributed among both groups, the isolated occurrence of visual defects was only seen in case of resistance.

**Fig. 1:**
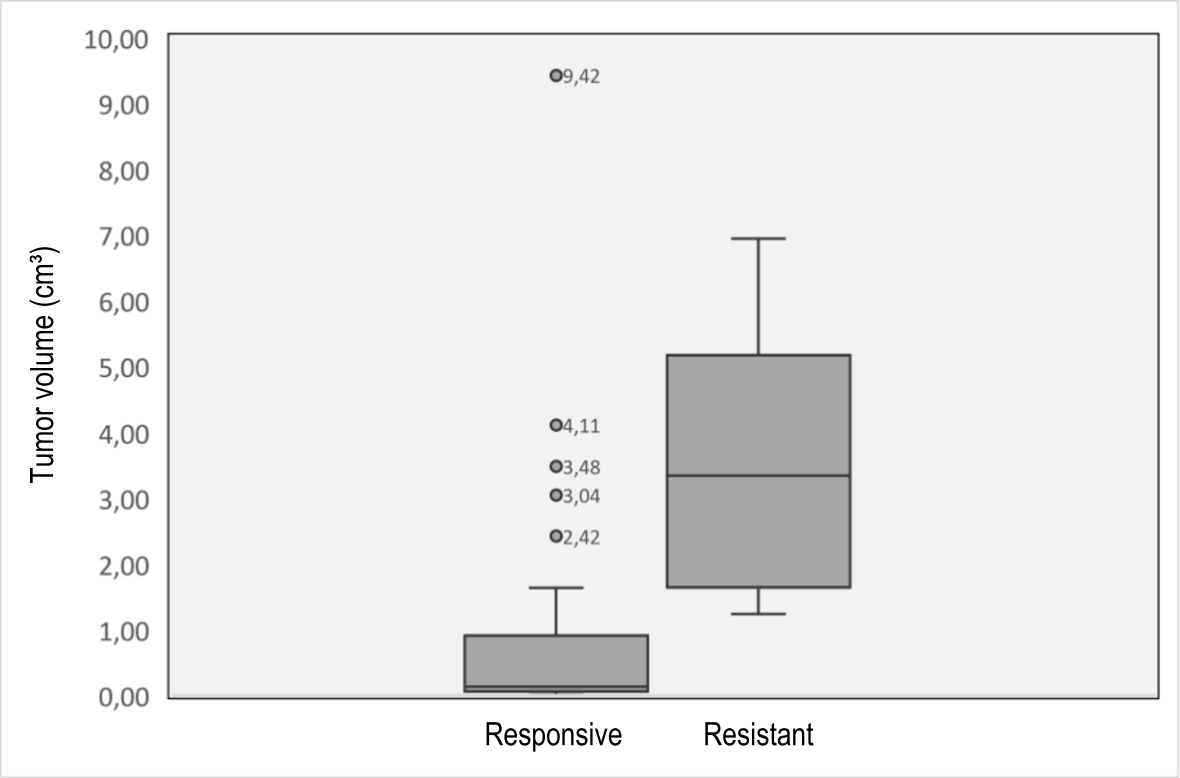
Comparison of tumour volume in the responsive and resistant patient subgroup (significantly higher in resistant prolactinomas, *p* = 0.015, Mood’s median test, 95% CI)

Time to prolactin normalization appeared to have a significant association with resistance (*p* = 0.008). Analyses of the MRI images of the pituitary gland showed an association between dopamine agonist resistance and tumour classification (micro- or macroadenoma). Here, 72.7% of the resistant tumours were macroadenoma, compared to 40.4% in the non-resistant group. The median tumour volume in the resistant group was 3.21 cm^3^ higher (*p* = 0.02) than in the group of patients responding well to the dopamine agonists (0.13 cm^3^)(Fig. 1).

Furthermore, significantly more patients in the dopamine agonist resistant group revealed the presence of a cystic, necrotic or hemorrhagic component on MRI (before the start of the pharmacological treatment) compared to the responsive group (71.43% versus 20.75% respectively; *p* = 0.004).

Regarding the tumour density factors, only contrast enhancement appeared to be a possible predictive factor for resistance to dopamine agonists (28% higher in the resistant group).

### Prediction model

Based on a linear discriminant analysis, we were able to statistically quantify the contribution of all factors in the prediction of resistance to the dopamine agonist cabergoline (Wilks lambda significance). It is noteworthy that baseline prolactin level did not appear to make a significant contribution to this prediction. Tumour volume and the classification of the tumour in micro- or macroadenoma are both significant predictors. Since these variables are clearly interrelated, we decided to only include the most powerful factor which turned out to be the tumour volume (Standardized Canonical Discriminant Function Coefficient of 0.381 versus 0.070).

**Fig. 2:**
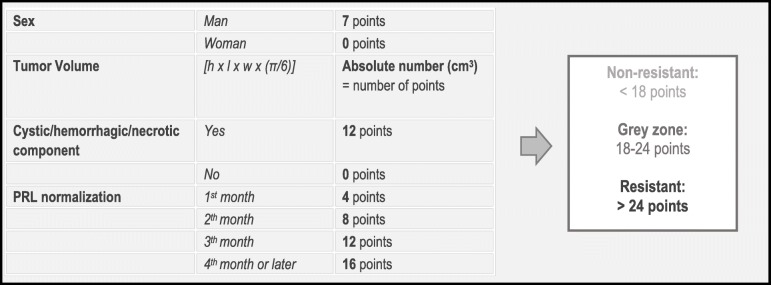
Scoring table (prediction model) based on 4 identified factors (sex, tumour volume, time to prolactin normalization and the presence of a cystic, hemorrhagic or necrotic component) to identify patients at risk for dopamine agonist resistant prolactinoma

After eliminating the weakest predictors, we came up with a strong model where 4 factors can determine the response to dopamine agonists with nearly 85% certainty. The 4 most powerful predictors are: sex, tumour volume, the moment of prolactin normalization and the presence of a cystic, hemorrhagic or necrotic component (before the start of the dopamine agonist treatment). These are scored using a specialized scoring system (Fig. 2). Weaker predictors such as the presence of visual defects, a high baseline prolactin level and a high contrast enhancement on MRI, can also be taken into account for the clinical decision. In our study population itself, 89.5% could be correctly classified on the basis of the scoring system if the cut-off of 20 points was considered.

## Discussion

In the present retrospective study, we thought to investigate possible predictive factors of pharmacological resistance of prolactinomas to dopamine agonists.

As expected, we found a higher general frequency of microprolactinomas compared to macroprolactinomas. There was a higher incidence of macroprolactinomas in men (88.2%) compared to women (30.8%). This trend is by analogy with previous literature [16–18].

The correlation we found between the (isolated) presence of visual defects and resistance was confirmed in literature, although they did not investigate the association with other combined mass effects such as headaches [15]. We have only found a significant difference between both groups when comparing patients who merely had visual defects at time of diagnosis.

In accordance with the guidelines of the Pituitary Society for the diagnosis and management of prolactinomas [5], 97% of our patients received dopamine agonists as first-line treatment for their prolactinoma. In these patients, we found 15.9% to be resistant to dopamine agonists treatment, which is in accordance with previous data on this issue in literature, reporting resistance rates between 10 to 18% [3, 6, 8, 19].

Subsequently, we have shown that male gender, a large tumour volume, prolonged time to prolactin normalization and presence of a cystic, hemorrhagic and/or necrotic component (before the start of the pharmacological treatment) had an important contribution in the prediction of resistance to dopamine agonists in prolactinoma patients. The results of our study extend previous studies.

The proposition that resistance to dopamine agonists is seen more frequently in men has previously been acknowledged in literature and is commonly adopted in clinical practice [16, 17]. Some authors believe that this is due to a higher incidence of macroadenomas in men. This higher incidence of macroadenomas in men could on the one hand be explained by the less obvious clinical symptoms compared to those in women [3]. On the other hand, Delgrange et al. postulated a different pathogenesesis in men compared to women. This is supported by the evidence that higher counts of cells with positive Ki67 proliferation markers were found in male patients (2.6 +/− 1.1% of positive nuclei) compared to female patients (versus 0.4 +/− 0.2% of positive nuclei) when prolactinomas of similar size were taken into consideration [20, 21]. This may explain an independent relationship between sex and resistance.

The strongest endocrinological predictor of resistance to dopamine agonists in our study appeared to be the time to prolactin normalization. Since there is a clearly defined dosage escalation protocol by Pfizer® in the tablet prescribing information, which is applied in most centers, time to prolactin normalization can be considered as a parameter. The association between the lack of prolactin normalization during medical therapy with a more aggressive evolution of prolactinoma and a higher proliferative potential, has already been reported [17, 21].

In contrast to what could have been expected from literature, we found no significant difference in baseline prolactin level between the two groups, nor did we find a predictive capacity for this factor [15, 17]. However, in a retrospective study of 74 patients who underwent transsphenoidal surgery, preoperative PRL levels did not correlate with the histological parameters (atypia and proliferation) [21].

Our study confirms previous studies, showing a correlation between resistance and the size of the tumour. Indeed, pharmacological resistant prolactinomas seem to be larger in our study (significant difference). However, the decrease in tumour volume could not be withheld as a valuable predictive factor [22]. A possible explanation may be that we did not have sufficient information about the evolution of the tumour volume. Data on the tumour volume after 3 months was only available in 17 of 69 patients. However, the presence of a cystic, hemorrhagic or necrotic component appears to have a significant value in predicting resistance to dopamine agonists. There are several authors who claim that prolactinomas with a cystic component are supposed to respond less to dopamine agonists [5, 9, 16]. Nevertheless, there are also studies in literature showing a response in terms of tumour size to DA in prolactinomas with a cystic component. It must be emphasized that the present study aims to look at the impact of DA both in terms of tumour size as well as prolactin level to define responsiveness. The contrast enhancement in resistant prolactinomas is significantly higher, despite the fact that this is a low-powered predictor for resistance. Previous research shows that resistant adenomas would have an increased angiogenesis, which could be an explanation for the increased enhancement [16]. Although this factor is not included in the predictive model, it can be taken into account in the grey area.

It is important to put our results into perspective because of a small study population. Although we have established the sample size for inclusion on the basis of a statistical power analysis, the accuracy of some statistical tests used in the analysis becomes withal less reliable in a small sample size. To further clarify, 91% of our patient population is treated with cabergoline causing that our model is mainly accurate for the most commonly used treatment. It is possible that there is a predictive value for other less frequently used dopamine agonists, but this is not investigated in our study and therefore uncertain.

Gonzaga et al. describe in a very recent study that 15–20% of the patients require a weekly dose higher than 2 mg of cabergoline [23]. Some studies claim that resistant prolactinomas do not exist since a response rate of 99.3% can be achieved with high dose cabergoline (3-12 mg/week). However, doses as high as these may carry some cardiologic risks such as valve regurgitation and fibrosis [13, 24].

We have provided a predictive model for dopamine resistance based on the identified predictive factors. If dopamine resistance is suspected, surgery could be offered as an alternative first-line treatment instead of medical treatment [25]. Indeed, remission rates of surgical resection as high as 91% have been reported especially in the case of microprolactinomas, and if performed by an experienced surgeon, the risk of complications remains relatively small (1,5%-6,5%) [2, 8].

## Conclusion

We retrospectively analyzed a rather limited although highly representative database of a Belgian tertiary reference centre for patients with pituitary tumours.

We developed a prediction model based on the 4 most powerful predictors of resistance to dopamine agonists being male gender, a great tumour volume, prolonged time to prolactin normalization and the existence of a cystic, hemorrhagic or necrotic component (before the start of the pharmacological treatment).

The scoring system is meant to be a tool to objectively evaluate the patient’s response to the dopamine agonists early in the course of treatment. In this way, patients who are at high risk of resistance can be identified early and operated before the fibrosis which is induced by long term dopamine agonist therapy, occurs. To compensate for the inaccuracy of this model, a grey zone was built in. Weaker predictors such as the presence of visual defects, a high baseline prolactin level and a high contrast enhancement on MRI, are factors that can be taken into account for further interpretation for patients scoring within that grey zone.

This scoring system may have impact on the timing and decision of surgery in prolactinoma patients after further prospective evaluation.

## Data Availability

The datasets used and/or analyzed during the current study are not publicly available due to privacy matters but are available from the corresponding author on reasonable request.

## Abbreviations

ACTH: Adrenocorticotropic hormone
BRC: Bromocriptine
CAB: Cabergoline
CSF: Cerebrospinal fluid
DA: Dopamine agonists
h x l x w: Height x length x width
IGF-I: Insulin like growth factor –I
IQR: Interquartile range
MRI: Magnetic resonance imaging
PRL: Prolactin
PV: Pixel value
TSH: Thyroid stimulating hormone
χ^2^ test: Chi-squared test
